# Increased risk of skin cancers in mucous membrane pemphigoid: a large-scale matched cohort study of 117 million US individuals

**DOI:** 10.3389/fmed.2025.1585167

**Published:** 2025-04-03

**Authors:** Amar Nouri, Henning Olbrich, Enno Schmidt, Ralf J. Ludwig, Philip Curman

**Affiliations:** ^1^Dermato-Venereology Clinic, Karolinska University Hospital, Stockholm, Sweden; ^2^Division of Dermatology and Venereology, Department of Medicine (Solna), Karolinska Institutet, Stockholm, Sweden; ^3^Department of Dermatology, University-Hospital Schleswig-Holstein, Lübeck, Germany; ^4^Lübeck Institute of Experimental Dermatology, University of Lübeck, Lübeck, Germany; ^5^Institute and Comprehensive Centre for Inflammation Medicine, University-Hospital Schleswig-Holstein, Lübeck, Germany; ^6^Department of Medical Epidemiology and Biostatistics, Karolinska Institutet, Stockholm, Sweden

**Keywords:** autoimmunity, autoimmune, Pemphigus, pemphigoid, skin cancer, cancer, matched cohort study, mucous membrane pemphigoid

## Abstract

**Introduction:**

Mucous membrane pemphigoid (MMP) is an autoimmune disease affecting the oral mucosa, conjunctivae and other mucous membranes. The mainstay treatment options are local and systemic corticosteroids and immunomodulatory therapies. Current research on cancer risk in MMP is scarce and has yielded conflicting results.

**Methods:**

The aim of this study was to investigate the risk of developing skin cancer in patients with MMP by performing a large-scale, retrospective matched cohort study utilizing data from over 117 million US individuals. The risk of skin cancer in patients with MMP was observed within a 5-year follow-up period, along with three temporal difference analyses and stratification for disease severity.

**Results:**

MMP was associated with an increased risk of several types of skin cancers within the first 5 years of follow-up, particularly squamous cell carcinoma, basal cell carcinoma, and non-melanoma skin cancer. Stratification by disease severity showed significantly elevated risks in severe cases.

**Discussion:**

These findings underscore the importance of regular skin cancer screening and risk-based monitoring in MMP patients, particularly those with severe disease. Integrating dermatologic surveillance into routine care could facilitate early detection and timely intervention. Additionally, these results highlight the need for further research into cancer risks in other autoimmune blistering diseases, helping to refine long-term management strategies.

## 1 Introduction

Mucous membrane pemphigoid (MMP) is a rare autoimmune blistering disease (AIBD) that causes erosions in the mouth, pharynx, larynx, esophagus, trachea, nose, genitalia and perianal area resulting in pain and strictures (1–5). In addition, conjunctival lesions lead to visual impairment and finally, blindness (6). In about a quarter of patients, skin lesions arise in addition to mucosal involvement. MMP is associated with autoantibodies that target components of the basement membrane zone attaching the epithelium/epidermis to the lamina propria/epidermis leading to its detachment and blister formation (4, 7, 8). The etiology of MMP is not yet fully understood and is likely rooted in a combination of genetic factors and various environmental triggers (7–9).

The mainstay treatment for mild MMP is local corticosteroids. More severe forms require treatment with systemic corticosteroids, anti-inflammatory agents, immunosuppressants, or intravenous immunoglobulins, among others (8, 10, 11). The most common autoantigen in MMP is BP180 (also known as type XVII collagen). In about 5% of MMP patients, autoantibodies react with type VII collagen, while anti-BP230 reactivity can be found in some patients with anti-BP180 antibodies. In individual MMP patients, IgG against α6β4 integrin can be detected (2, 7–10).

Current insights on the topic of MMP and cancer are limited in scope and often yield conflicting results (12–17). Limitations include low sample sizes, lack of adequate control groups, and imperfect methodological rigor. The association between anti-laminin 332 reactivity and malignancies, mostly solid cancers, has been clearly established in recent studies following the original descriptions by Leverkus et al. and Egan et al. (13, 18–23). This observation was facilitated by the development of standardized, sensitive, and specific detection systems for serum anti-laminin 332 IgG (20, 24). Consequently, national and international guidelines recommend testing for circulating anti-laminin 332 IgG in newly diagnosed MMP patients (2, 4, 10). The mechanisms responsible for the cancer risk in patients with MMP are not yet fully understood and large-scale studies investigating the possible correlation between cancer and MMP are lacking. Using the extensive TriNetX Analytics Network, this study set out to specifically examine the relationship between MMP and skin cancer.

## 2 Materials and methods

### 2.1 Study design and database

**FIGURE 1:**
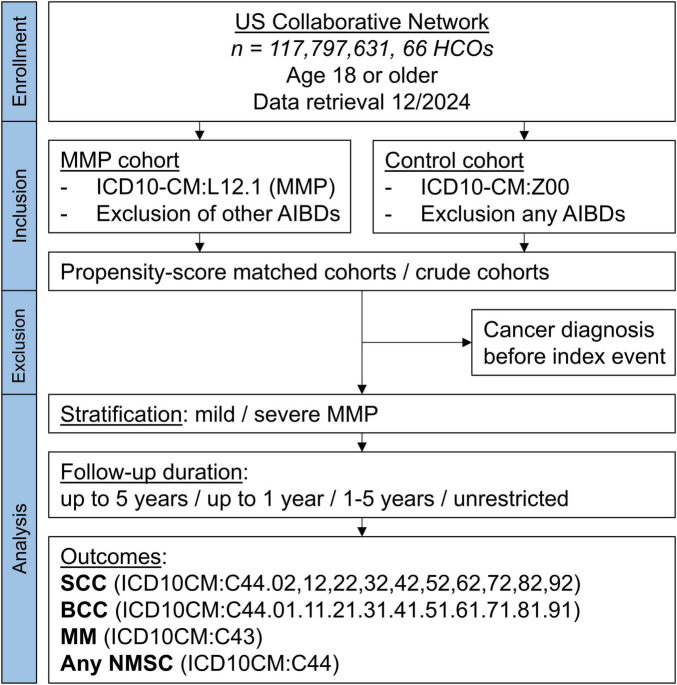
Study flow chart. AIBD, autoimmune blistering disease; BCC, basal cell carcinoma; HCO, health care organization; MM, malignant melanoma; MMP, mucous membrane pemphigoid; NMSC, non-melanoma skin cancer.

Electronic health record (EHR) data from the US Collaborative Network of the federated TriNetX platform was used in this large-scale propensity-score matched cohort study, following previously used designs (25–27). TriNetX provides secure, computerized access to EHRs in real-time (28). Data from almost 118 million EHRs in the US Collaborative Network was sampled and retrieved in December of 2024 (Figure 1).

A cohort of individuals with a diagnosis of MMP was defined and compared with individuals without a diagnosis of MMP. The start of the study period, marking the index event and entry of each participant in the study, was defined as a diagnosis of MMP for cases and a diagnosis of ICD-10CM:Z00 “Encounter for general examination without complaint, suspected or reported diagnosis” for controls. Only individuals aged 18 years and older were included.

### 2.2 Cohort definitions and subgroup analyses

A total of 3,812 patients with MMP were enrolled in the study. The patient cohort was defined by the inclusion of an ICD-10CM code L12.1 and the exclusion of the following codes: L10, L11, L12.0, L13, and L14 (other AIBDs), to ensure a high probability of defining a cohort truly consisting of MMP patients. Controls (*n* = 12,777,220) were defined by ICD-10CM:Z00 and by the exclusion of any AIBD L10-L14. In addition, a subgroup analysis for mild and severe MMP was performed. Severe MMP was defined as the prescription of any systemic drug commonly known to be used in the treatment of MMP (Supplementary Table 1), while mild MMP was defined as the exclusion of any such medications except prednisone and tetracyclines. Only medications from the time of diagnosis were included. The index event, marking the start of the study and follow-up, was defined by the time of diagnosis.

The study outcomes were squamous cell carcinoma (SCC) of the skin, basal cell carcinoma (BCC), malignant melanoma (MM), and any non-melanoma skin cancer (NMSC) (Supplementary Table 2).

### 2.3 Covariates

A crude analysis was performed, not accounting for any covariates, as well as rigorous propensity-score matching (PSM) to balance the cohorts and optimize comparability. A covariate matrix for PSM was established, including demographic information and potentially influential confounders, including the following covariates: age at index (continuous variable), white race (binary), female sex (binary), personal history of nicotine dependence (ICD-10CM:Z87.891, binary), nicotine dependence (ICD-10CM:F17, binary), and overweight and obesity (ICD-10CM:E66, binary), and family history of primary malignant neoplasm (ICD-10CM:Z80, binary). In addition, a large number of demographic variables and relevant comorbidities were extracted for the baseline characteristics in Table 1 but not matched for. The matrix row order was randomized after data retrieval. A propensity-score for each patient was generated by logistic regression analysis (with exposure as the dependent variable) using the Python package scikit-learn. Matching was performed for cases: controls on a 1:1 ratio using the greedy nearest neighbor approach with a cut-off distance of 0.1 pooled standard deviations of the logit of the propensity-score. Baseline characteristics were re-evaluated and reported after matching, differences were compared by *t*-test for continuous and *z*-test for binary or categorical variables.

**TABLE 1:** Baseline characteristics of participants in the primary analysis before and after propensity-score matching.

Patient characteristics								
**Participant data**	**–**	**Cohort**	**Before matching**			**After matching**		
Number of participants	**–**	MMP	3,812			3,723		
		Control	12,777,229			3,723		
Mean follow-up time (days)	**–**	MMP	896			896		
		Control	1,074			1,103		
**Demographic and clinical data**	**ICD-10CM code**	**Cohort**	**Patients**	***P*-value**	**Std diff.**	**Patients**	***P*-value**	**Std diff.**
Age at index (*n*, years +/- SD)*	**-**	MMP	3,723 (64.0+/**-**13.9)	<0.001	1.093	3,723 (64.0+/**-**13.9)	0.973	0.001
		Control	12,488,052 (46.5+/**-**17.9)			3,723 (64.0+/**-**13.9)		
BMI (mean +/- SD)**	**-**	MMP	29.0+/**–**7.2	0.710	0.010	29.0+/**-**7.2	0.435	0.028
		Control	29.1+/**-**7.2			29.2+/**-**6.7		
Patients with registered BMI value (*n*, %)	**-**	MMP	1,288 (34.6)	<0.001	0.173	1,288 (34.6)	< 0.001	0.282
		Control	5,369,367 (43.0)			1,801 (48.4)		
White (*n*, %)*	**-**	MMP	2,759 (74.1)	<0.001	0.242	2,759 (74.1)	0.833	0.005
		Control	7,861,783 (63.0)			2,751 (73.9)		
Female (*n*, %)*	**-**	MMP	2,329 (62.6)	<0.001	0.183	2,329 (62.6)	0.962	0.001
		Control	6,687,449 (53.6)			2,327 (62.5)		
Personal history of nicotine dependence (*n*, %)*	Z87.891	MMP	286 (7.7)	<0.001	0.089	286 (7.7)	0.793	0.006
		Control	684,335 (5.5)			280 (7.5)		
Nicotine dependence (*n*, %)*	F17	MMP	164 (4.4)	<0.001	0.092	164 (4.4)	0.822	0.005
		Control	810,863 (6.5)			168 (4.5)		
Family history of primary malignant neoplasm (*n*, %)*	Z80	MMP	190 (5.1)	0.015	0.038	190 (5.1)	0.594	0.012
		Control	536,529 (4.3)			180 (4.8)		
Overweight and obesity (*n*, %)*	E66	MMP	354 (9.5)	0.124	0.026	354 (9.5)	0.937	0.002
		Control	1,283,009 (10.3)			352 (9.5)		
Essential (primary) hypertension (*n*, %)	I10	MMP	876 (23.5)	<0.001	0.057	876 (23.5)	< 0.001	0.276
		Control	2,642,723 (21.2)			1,342 (36.0)		
Hyperlipidemia, unspecified (*n*, %)	E78.5	MMP	609 (16.4)	<0.001	0.074	609 (16.4)	< 0.001	0.196
		Control	1,712,653 (13.7)			901 (24.2)		
Type 1 diabetes mellitus (*n*, %)	E10	MMP	56 (1.5)	0.026	0.034	56 (1.5)	0.430	0.018
		Control	139,872 (1.1)			48 (1.3)		
Type 2 Diabetes mellitus (*n*, %)	E11	MMP	380 (10.2)	0.007	0.043	380 (10.2)	< 0.001	0.137
		Control	1,117,888 (9.0)			548 (14.7)		
Ischemic heart disease (*n*, %)	I20**-**I25	MMP	310 (8.3)	<0.001	0.106	310 (8.3)	< 0.001	0.099
		Control	703,611 (5.6)			420 (11.3)		
Cerebral infarction (*n*, %)	I63	MMP	99 (2.7)	<0.001	0.069	99 (2.7)	0.828	0.005
		Control	206,698 (1.7)			96 (2.6)		
Chronic kidney disease (*n*, %)	N18	MMP	153 (4.1)	0.028	0.035	153 (4.1)	< 0.001	0.129
		Control	430,876 (3.5)			263 (7.1)		
Fibrosis and cirrhosis of the liver (*n*, %)	K74	MMP	30 (0.8)	0.207	0.019	30 (0.8)	0.533	0.014
		Control	80,003 (0.6)			35 (0.9)		
Persons with potential health hazards related to socioeconomic and psychosocial circumstances (*n*, %)	Z55**-**Z65	MMP	96 (2.6)	0.629	0.008	96 (2.6)	0.550	0.014
		Control	338,077 (2.7)			88 (2.4)		

MMP, mucous membrane pemphigoid; BMI, Body Mass Index; ICD-10CM, International Classification of Diseases 10th edition Clinical Modification; PSM, propensity-score matching; SD, standard deviation.

*Variables included in PSM.

**BMI was only recorded for 35% of patients and 43% of controls. Values in bold mark statistically significant results.

### 2.4 Statistical analysis

The primary analysis investigated outcomes for crude and matched analyses 1 day to 5 years after index. To test the robustness of the results in the primary analysis, three matched analyses on temporal differences were performed on all MMP: (1) only outcomes 1 day to 1 year after index were considered to examine short-term effects, (2) outcomes 1-5 years after index were analyzed to reduce the potential influence of detection bias and reverse causality, and (3) outcomes 1 day to any time after index were examined for long-term associations. Any outcomes prior to the index event were excluded in all analyses. The subgroup analysis for mild and severe MMP was performed only for the primary follow-up time of 5 years.

Risk ratios, odds ratios, and risk differences were calculated. Survival analyses were performed using Kaplan–Meier (KM) analysis. The proportionality assumption was tested by the coxph function in R’s Survival package using Schoenfeld residuals and χ^2^ tests. KM-curves were compared using the Log-rank test. A univariate Cox proportional hazards regression was used to express hazard ratios (HR)s with 95%-confidence intervals (CI)s.

### 2.5 Ethics statement

TriNetX data is presented solely in aggregated form and only contains anonymized data complying with the de-identification standard as defined by the US Health Insurance Portability and Accountability Act (HIPAA) in section §164,514(a). TriNetX is certified to the ISO 27001:2013 standard and maintains a so-called Information Security Management System to ensure rigorous protection of the healthcare data it has access to, following the HIPAA Security Rule. In addition, the Swedish Ethical Review Authority has granted ethical approval for this study (diary number 2024-06878-02).

## 3 Results

### 3.1 Study population characteristics

Data from a total of 117,797,631 patients from 66 health care organizations were screened (Figure 1). The number of participants after successful PSM was 3,723 MMP patients and an equal number of controls. No significant differences were found in any of the covariates after PSM (Table 1).

### 3.2 Increased risk of skin cancers in MMP

**FIGURE 2:**
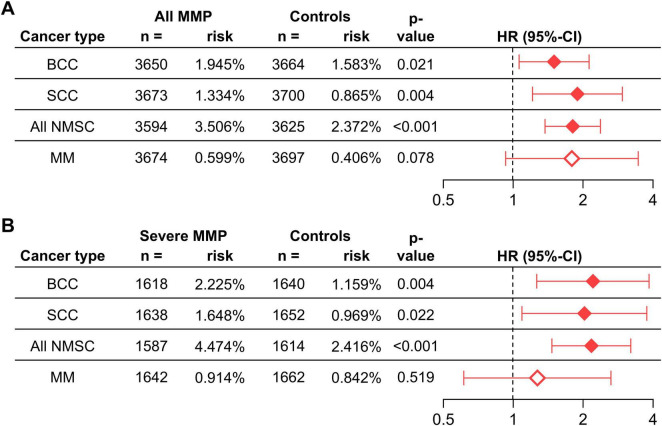
The risk of skin cancers in individuals with any MMP **(A)** and severe MMP **(B)** compared with controls without MMP. BCC, basal cell carcinoma; CI, confidence interval; HR, hazard ratio; MM, malignant melanoma; MMP, mucous membrane pemphigoid; NMSC, non-melanoma skin cancer.

MMP was associated with a significantly increased risk of several types of skin cancers within the first 5 years of follow-up (Figure 2) and more so in the crude analysis (Supplementary Table 3). The highest risk was observed for SCC which was more than tripled in the crude analysis and almost doubled in the matched analysis (HR 1.892, 95% CI 1.211–2.955, *p* = 0.004). An increased risk was also seen for BCC in the crude and matched analysis (HR 1.501, 95% CI 1.061–2.125, *p* = 0.021). Diagnosed MMs showed a significantly increased risk in the crude analysis, but not in the matched analysis. The risk of any NMSC was significantly increased in the crude and matched analysis (HR 1.807, 95% CI 1.373–2.378, *p* < 0.001) (Figure 2 and Supplementary Table 3). Since the risk of skin cancer, especially melanoma, is known to be increased in individuals with a history of previous skin cancers (29), an analysis with the addition of “Personal history of malignant neoplasm of skin” (ICD-10CM:Z85.82) as a covariate for PSM was performed, showing no significant change in risk relationships (results not shown).

### 3.3 Subgroup analysis for disease severity in MMP

When stratifying the analysis by disease severity for outcomes with a 5-year follow-up, significant risk increases for mild MMP were only found for SCC, BCC, and NMSC in the crude analysis but not for any outcome in the matched analysis (Supplementary Tables 3, 4). Interestingly, the observed risks were substantially augmented in patients with severe MMP. SCC showed an almost 4 times increased risk in the crude analysis, and the risk was doubled in the matched analysis (HR 2.026, 95% CI 1.091–3.763, *p* = 0.022). Risk increases for BCC were 2.5 in the crude and 2.2 times in the matched analysis (HR 2.206, 95% CI 1.264–3.848, *p* = 0.004). MM displayed a significantly increased risk in the crude analysis but was not significant in the matched analysis. Any NMSC, however, had an almost tripled risk in the crude analysis, and doubled risk in matched analysis (HR 2.171, 95% CI 1.468–3.211, *p* < 0.001) (Figure 2 and Supplementary Table 3).

### 3.4 Temporal differences

All outcomes were analyzed for a 1-year follow-up with matched analyses. A significant risk was observed for SCC (HR 2.339, 95% CI 1.009–5.420, *p* = 0.041), while BCC and MM showed trends towards increased risks, albeit not significant. Significant risks remained for any NMSC (HR 1.710, 95% CI 1.137–2.578, *p* = 0.009). When analyzing for a 1–5-year follow-up, there were no significant increased risks for SCC, BCC, or MM. However, there was a significant increase in any NMSC in this group (HR 1.594, 95% CI 1.115–2.277, *p* = 0.010). When extending the follow-up to any time after index, significant risk increases for SCC (HR 1.594, 95% CI 1.115–2.277, *p* = 0.010), MM (HR 1.665, 95% CI 1.015–2.731, *p* = 0.041), and any NMSC (HR 1.492, 95% CI 1.184–1.880, *p* = 0.001) were observed, while BCC showed no significance (Table 2).

**TABLE 2:** Propensity-score matched analyses of temporal differences in patients with mucous membrane pemphigoid.

Temporal differences				
**Outcome**	**Cohort**	Eligible participants* **(nr with outcome)**	**HR (CI)**	***P*-value**
**0-1 years**				
Squamous cell carcinoma	MMP	3,673 (17)	**2.339 (1.009, 5.420)**	**0.041**
	Control	3,696 (10**)		
Basal cell carcinoma	MMP	3,650 (36)	1.532 (0.925, 2.538)	0.095
	Control	3,657 (26)		
Melanoma	MMP	3,674 (10**)	1.985 (0.665, 5.924)	0.210
	Control	3,706 (10**)		
Any non-melanoma skin cancer	MMP	3,594 (59)	**1.710 (1.137, 2.570)**	**0.009**
	Control	3,600 (38)		
**1-5 years**				
Squamous cell carcinoma	MMP	3,656 (32)	1.546 (0.926, 2.581)	0.093
	Control	3,688 (27)		
Basal cell carcinoma	MMP	3,614 (35)	1.424 (0.881, 2.300)	0.147
	Control	3,631 (32)		
Melanoma	MMP	3,665 (13)	0.936 (0.458, 1.910)	0.855
	Control	3,701 (18)		
Any non-melanoma skin cancer	MMP	3,535 (67)	**1.594 (1.115, 2.277)**	**0.010**
	Control	3,562 (55)		
**Any time**				
Squamous cell carcinoma	MMP	3,673 (62)	**1.548 (1.076, 2.227)**	**0.018**
	Control	3,696 (56)		
Basal cell carcinoma	MMP	3,650 (87)	1.293 (0.962, 1.739)	0.087
	Control	3,657 (91)		
Melanoma	MMP	3,674 (35)	**1.665 (1.015, 2.731)**	**0.041**
	Control	3,706 (29)		
Any non-melanoma skin cancer	MMP	3,594 (153)	**1.492 (1.184, 1.880)**	**<0.001**
	Control	3,600 (139)		

MMP, mucous membrane pemphigoid; HR, hazard ratio; CI, confidence interval.

*Participants with the outcome prior to index were excluded.

**Patient counts below 10 are suppressed in the database to protect patient confidentiality. Values in bold mark statistically significant results.

## 4 Discussion

A significant association between MMP and specific skin cancers, particularly SCC, BCC, and total NMSC, was found in this large-scale propensity-score matched cohort study. The findings highlighted that the risks were most pronounced in patients with severe forms of MMP.

The mechanisms responsible for the risk of cancer in patients with MMP are not yet fully understood, with some research suggesting that the associated serotypes may confer anything from increased to reduced risk to having no influence. Most studies have focused on anti-laminin 332 MMP, despite it being a minority among MMP patients (12). Evidence primarily suggests an increased risk of internal malignancies, particularly adenocarcinomas (12–14, 16), with this risk potentially being higher within the first year of blister onset (13). Other forms of MMP include serotypes which produce antibodies against the different subunits of the α6β4-integrin heterodimer, which instead is thought to be linked to a decreased risk of cancer (15, 16), and a serotype with production of antibodies against BP180 and BP230 which seems to have no effect on cancer risk (12, 16).

The role of different antigens regarding tumor development, survival, proliferation, invasion, and prognosis is complex. Some research has shown that the expression of laminin 332 is increased in some tumor cell lines, while being decreased in others. However, it is generally believed that laminin 332 in most cases acts as a tumor suppressant by promoting tissue homeostasis, which would explain the increased risk of cancer in patients with anti-laminin 332 MMP (13). Some studies even suggest a direct correlation between tumor burden and disease severity (14), with some cases even reporting significant clinical improvement of the disease after successful treatment of the primary tumor (13, 18, 30, 31). In contrast, the α6β4 heterodimer is most often associated with promoting tumor growth and spread. This might explain why patients with anti-α6β4 integrin MMP show a decreased risk of cancer in some studies. Interestingly, the α6 and β4-integrin antibody titers seem to be correlated with disease activity and decrease as the symptoms improve and may even become negative with remission (16, 32–34). Of note, only individual MMP patients were shown to react with α6β4 integrin, and no reliable test system is available for these autoantibodies. While the main target antigen of MMP is BP180, the presence of these antibodies did not correlate with disease activity or severity or an increased risk of malignancy, which is why it can be believed to be a secondary phenomenon (12, 16).

Patients with MMP, especially individuals with more severe forms of the disease, are likely to have a higher level of autoimmunity compared to individuals of the general population, which could speculatively lead to an increased risk of cancer. This, coupled with a higher degree of inflammation causing long-term tissue damage and requiring constant cellular changes and repair, is likely to lead to the promotion of malignant transformation. Most medications used as treatment of severe MMP are immunosuppressives that reduce immune surveillance and weaken the immune system’s ability to detect and eliminate cancerous cells. However, this risk typically manifests after a timeline of more than 3-5 years (35, 36) and because of this, the elevated cancer prevalence observed in severe MMP cannot be explained solely by the effects of these treatments.

Overall different risk relationships were seen between NMSC and MM. Possible explanations might be that both MMP and NMSC involve dysfunction of the basement membrane zone and share autoimmune mechanisms affecting epithelial integrity (33, 37), while MM develops from melanocytes, being less affected by the autoimmune process in MMP compared to keratinocytes. Second, it could be speculated that the prevalence of MM in patients with more severe forms of MMP is higher due to broader immune system dysfunction that affects melanoma surveillance.

The present study described patients with MMP to be at an increased risk of developing skin cancer, particularly SCC, BCC, and NMSC, with the risks being most pronounced in severe cases. This underscores the importance of implementing tailored follow-up protocols and regular cancer screenings for MMP patients to facilitate early detection and timely intervention. Personalized care strategies that account for disease severity and cancer risk could significantly improve patient outcomes. Furthermore, these findings highlight the potential value of investigating similar cancer risks in other autoimmune blistering diseases and, more broadly, across autoimmune disorders. Such research could contribute to a deeper understanding of the complex interplay between chronic inflammation, autoimmunity, and cancer development.

The main strength of this study lies in the use of a large and diverse cohort, allowing for detailed propensity-score matching (PSM) to minimize confounding bias. The extensive sample size enhances the generalizability of our findings, and the inclusion of rigorous temporal differences across various follow-up periods strengthens the robustness of the results. The large sample size also allows for a comparison of mild and severe MMP, emphasizing the correlation to disease severity. This comprehensive approach provides valuable insights into cancer risks in patients with MMP, making the findings relevant for clinical practice.

A limitation of the study is the absence of specific autoantibody data, such as anti-laminin 332, which prevented examination into potential differences between MMP subtypes. However, since subtype testing is not routine in most clinical settings, the findings still offer a broad and clinically meaningful understanding of cancer risk in MMP. Additionally, although detection bias is a potential concern, given that MMP patients might undergo more frequent monitoring near diagnosis, analysis of temporal differences revealed this impact is likely small. While PSM controlled for many important variables, residual confounding from unmeasured factors such as UV radiation therapy and immunosuppressive post-MMP diagnosis, remains possible. The use of medications as a proxy for severity stratification could also constitute a limitation, as exposure to these may confound skin cancer risk. While statistically significant, the sometimes wide confidence intervals warrants caution when interpreting the strengths of the observed risk associations. Finally, the retrospective design and use of ICD-10CM codes introduces the risk of miscoding and misclassification, as well as limits causal inferences.

This study demonstrates a significant correlation between MMP and SCC, BCC, and NMSC in general, and in severe cases in particular. While no correlation was found between MMP and MM in the 5-year follow-up for mild cases, significant associations were observed in severe MMP and in longer-term analyses. Current research regarding the risk of cancer in different MMP subtypes provides conflicting results, and these relationships likely involve complex mechanisms including autoimmunity and inflammation. Highlighting these risks is crucial for improving awareness and early detection. Future research is warranted to explore the underlying mechanisms driving these associations, including the roles of chronic inflammation, immune dysregulation, and potential genetic predispositions.

## Data Availability

The data analyzed in this study is subject to the following licenses/restrictions: Specific institutional accesses. Requests to access these datasets should be directed to PC, philip.curman@ki.se.
