# Supplementary material for: Increased risk of skin cancers in mucous membrane pemphigoid: a large-scale matched cohort study of 117 million US individuals
Source: Front Med (Lausanne). 2025 Apr 3;12:1585167. doi: 10.3389/fmed.2025.1585167 (PMC12003376; doi:10.3389/fmed.2025.1585167)
Supplement: Supplementary file 1 [file Table_1.docx]

**Supplementary tables**

**Supplementary table 1.** All medications used as proxies for severity stratification in mucous membrane pemphigoid.

- Azathioprine
- Cyclophosphamide
- Cyclosporine
- Dapsone
- Dupilumab
- Immunoglobulin G
- Leflunomide
- Methotrexate
- Methotrexate sodium
- Mycophenolate (mycophenolic acid)
- Mycophenolate acyl-glucuronide
- Mycophenolate glucuronide
- Mycophenolate mofetil
- Omalizumab
- Rituximab
- Rituximab Ab
- Rituximab and hyaluronidase
- Rituximab-pvvr, biosimilar, (ruxience)
- Rituximab-abbs, biosimilar, (truxima)
- Rituximab-arrx, biosimilar, (riabni)
- Thalidomide
- Sulfasalazine

**Supplementary table 2.** Study outcome definitions according to ICD-10CM.

**Squamous cell carcinoma**

Either of the following:

- C44.02
- C44.12
- C44.22
- C44.32
- C44.42
- C44.52
- C44.62
- C44.72
- C44.82
- C44.92

**Basal cell carcinoma**

Either of the following:

- C44.01
- C44.11
- C44.21
- C44.31
- C44.41
- C44.51
- C44.61
- C44.71
- C44.81
- C44.91

**Melanoma**

- C43

**Any non-melanoma skin cancer**

- C44

**Supplementary table 3.** Crude analyses for patients with mucous membrane pemphigoid. *Note: due to the data analysis being conducted at different time points, the number of study participants varies slightly between the crude and matched analyses.*

CI: Confidence Interval; HR: Hazard Ratio; MMP: Mucous Membrane Pemphigoid.

*Participants with the outcome prior to index were excluded. **Patient counts below 10 are suppressed in the database to protect patient confidentiality.

| **Primary analysis (0-5 years)** | | | |
| --- | --- | --- | --- |
| **All MMP** | | | |
|  |  | **Crude** | |
| **Outcome** | **Cohort** | **Eligible participants* (nr with outcome)** | **HR (CI)** |
| **Squamous cell carcinoma** | MMP | 3,581 (46) | **3.665 (2.745, 4.894)** |
|  | Control | 12,192,794 (51,069) |  |
| **Basal cell carcinoma** | MMP | 3,558 (65) | **2.737 (2.146, 3.491)** |
|  | Control | 12,147,115 (96,721) |  |
| **Melanoma** | MMP | 3,585 (12) | **1.834 (1.041, 3.230)** |
|  | Control | 12,191,012 (26,113) |  |
| **Any non-melanoma skin cancer** | MMP | 3,514 (99) | **2.931 (2.407, 3.570)** |
|  | Control | 12,088,478 (139,576) |  |
|  |  |  |  |
| **Mild MMP** | | | |
|  |  | **Crude** | |
| **Outcome** | **Cohort** | **Eligible participants* (nr with outcome)** | **HR (CI)** |
| **Squamous cell carcinoma** | MMP | 1,991 (14) | **2.362 (1.399, 3.989)** |
|  | Control | 12,192,794 (51,069) |  |
| **Basal cell carcinoma** | MMP | 1,979 (25) | **2.216 (1.497, 3.279)** |
|  | Control | 12,147,115 (96,721) |  |
| **Melanoma** | MMP | 1,995 (10**) | 1.287 (0.483, 3.429) |
|  | Control | 12,191,012 (26,113) |  |
| **Any non-melanoma skin cancer** | MMP | 1,953 (36) | **2.238 (1.615, 3.103)** |
|  | Control | 12,088,478 (139,576) |  |
|  |  |  |  |
| **Severe MMP** | | | |
|  |  | **Crude** | |
| **Outcome** | **Cohort** | **Eligible participants* (nr with outcome)** | **HR (CI)** |
| **Squamous cell carcinoma** | MMP | 1,600 (23) | **3.853 (2.560, 5.799)** |
|  | Control | 12,192,794 (51,069) |  |
| **Basal cell carcinoma** | MMP | 1,587 (29) | **2.570 (1.786, 3.698)** |
|  | Control | 12,147,115 (96,721) |  |
| **Melanoma** | MMP | 1,610 (10**) | **2.561 (1.280, 5.121)** |
|  | Control | 12,191,012 (26,113) |  |
| **Any non-melanoma skin cancer** | MMP | 1,561 (46) | **2.875 (2.153, 3.838)** |
|  | Control | 12,088,478 (139,576) |  |

**Supplementary table 4.** Propensity score matched analyses for mild mucous membrane pemphigoid. *Note: due to the data analysis being conducted at different time points, the number of study participants varies slightly between the crude and matched analyses.*

CI: Confidence Interval; HR: Hazard Ratio; MMP: Mucous Membrane Pemphigoid.

*Participants with the outcome prior to index were excluded. **Patient counts below 10 are suppressed in the database to protect patient confidentiality.

| **Primary analysis (0-5 years)** | | | | |
| --- | --- | --- | --- | --- |
| **Mild MMP** | | | | |
|  |  | **Matched** | | |
| **Outcome** | **Cohort** | **Eligible participants* (nr with outcome)** | **HR (CI)** | **P-value** |
| **Squamous cell carcinoma** | MMP | 2,047 (15) | 1.044 (0.534, 2.043) | 0.900 |
|  | Control | 2,060 (20) |  |  |
| **Basal cell carcinoma** | MMP | 2,038 (23) | 0.914 (0.538, 1.553) | 0.740 |
|  | Control | 2,040 (34) |  |  |
| **Melanoma** | MMP | 2,048 (10**) | 1.345 (0.471, 3.843) | 0.578 |
|  | Control | 2,066 (10**) |  |  |
| **Any non-melanoma skin cancer** | MMP | 2,005 (40) | 1.033 (0.683, 1.561) | 0.879 |
|  | Control | 2,011 (52) |  |  |
